# Prenatal exposure to perfluoroalkyl and polyfluoroalkyl substances and childhood atopic dermatitis: a prospective birth cohort study

**DOI:** 10.1186/s12940-018-0352-7

**Published:** 2018-01-17

**Authors:** Qian Chen, Rong Huang, Li Hua, Yifeng Guo, Lisu Huang, Yanjun Zhao, Xia Wang, Jun Zhang

**Affiliations:** 1 Ministry of Education-Shanghai Key Laboratory of Children’s Environmental Health, Xinhua Hospital, Shanghai Jiao Tong University School of Medicine, 1665 Kongjiang Road, Shanghai, 200092 China; 2 Department of Pediatric Pulmonology, Xinhua Hospital, Shanghai Jiao Tong University School of Medicine, Shanghai, 200092 China; 3 Department of Dermatology, Xinhua Hospital, Shanghai Jiao Tong University School of Medicine, Shanghai, 200092 China; 4 Department of Pediatrics, Xinhua Hospital, Shanghai Jiao Tong University School of Medicine, Shanghai, 200092 China; 5 Clinical research unit, Xinhua Hospital, Shanghai Jiao Tong University School of Medicine, Shanghai, 200092 China; 6 Department of Child Health Care, Shanghai Children’s Hospital, Shanghai Jiao Tong University School of Medicine, Shanghai, 200040 China

**Keywords:** Perfluoroalkyl and polyfluoroalkyl substances, Cord blood, Exposure, Atopic dermatitis, Childhood

## Abstract

**Background:**

Perfluoroalkyl and polyfluoroalkyl substances (PFASs) have been reported to suppress immune function. However, previous studies on prenatal exposure to PFASs and allergic disorders in offspring provided inconsistent results. We aimed to examine the association between prenatal exposure to PFASs and childhood atopic dermatitis (AD) in offspring up to 24 months of age.

**Methods:**

A prospective birth cohort study involving 1056 pregnant women was conducted in two hospitals in Shanghai from 2012 to 2015. Prenatal information was collected by an interview with the women and from medical records. Fetal umbilical cord blood was collected at birth. Cord blood plasma PFASs were measured. Children were followed at 6, 12 and 24 months and information on the development of AD was recorded. AD was diagnosed by 2 dermatologists independently based on the questionnaires. Multiple logistic regression was used to compute odds ratio (OR) and corresponding 95% confidence interval (CI) for the association between AD and each PFASs, adjusting for potential confounders**.**

**Results:**

A total of 687 children completed a 2-year follow-up visit and had PFASs measurement. AD was diagnosed in 173 (25.2%) children during the first 24 months. In female children, a log-unit increase in perfluorooctanoic acid (PFOA) was associated with a 2.1-fold increase in AD risk (AOR 2.07, 95% CI 1.13–3.80) after adjusting for potential confounders. The corresponding risk was 2.22 (1.07–4.58) for perfluorononanoic acid (PFNA). The highest PFOA quartile was significantly associated with AD (2.52, 1.12–5.68) compared with the lowest quartile. The highest quartile of PFNA, perfluorodecanoic acid (PFDA) and perfluorohexane sulfonic acid (PFHxS) were associated with AD with AOR (95% CI) being 2.14 (0.97–4.74), 2.14 (1.00–4.57), and 2.30 (1.03–5.15), respectively. Additionally, the second quartile of perfluorododecanoic acid (PFDoA) was associated with a 3.2-fold increase in AD risk (3.24, 1.44–7.27). However, no significant associations were found in male children.

**Conclusions:**

Prenatal exposure to PFOA, PFDA, PFDoA and PFHxS significantly increased the risk of childhood AD in female children during the first 24 months of life. In addition, the associations between AD with prenatal exposure to PFNA were close to statistical significance.

**Electronic supplementary material:**

The online version of this article (10.1186/s12940-018-0352-7) contains supplementary material, which is available to authorized users.

## Background

Perfluoroalkyl and polyfluoroalkyl substances (PFASs) are synthetic compounds with strong fluorine-carbon covalent bonds. PFASs have strong thermal, biological, and chemical stability as well as hydrophobic and lipophobic properties, and have been widely used worldwide in industrial (lubricants, surfactants) and consumer products such as non-stick cookware, waxes, paints, cosmetics and as water and oil repellents for leather, paper, and textiles since 1950s [1]. Most of the PFASs members are resistant to degradation and bio-accumulative and are ubiquitous in environment such as air, water, sediment and sludge; as well as wildlife [2]. The half-lives in human bodies (t1/2) of three common PFASs, perfluorooctanoic acid (PFOA), perfluorooctane sulfonate (PFOS) and perfluorohexane sulfonate (PFHxS), are 3.8, 5.4 and 8.5 years, respectively [3]. Although the production of PFOA and PFOS and related compounds has been phased out in some countries, the production of these chemicals or their precursors has actually increased in Asia, especially in China [4–6]. Environmental exposure to PFASs has been of increasing public health concern.

Extensive researches have associated exposure to PFASs with immunotoxicity, hepatotoxicity, developmental toxicity, reproductive toxicity, neurotoxicity, endocrine toxicity, liver tumor, impaired thyroid function, and mammary glands development [7–11]. Experimental studies suggested that PFOA and PFOS may cause immune suppression at doses of daily exposure in human and animals [12, 13]. However, findings from epidemiologic studies were far from consistent [14, 15]. Furthermore, these were cross-sectional or case-control studies, and evidence of PFASs causing allergic disorders is still limited, particularly from prospective studies.

Atopic dermatitis (AD) is a skin disease due to skin barrier dysfunction, resulting in eczematous and itchy lesions at the flexural folds and other typical distributions [16]. It is often caused by a combination of immune dysfunction and environment. Most AD occurs before 1 year of age, and it is generally considered as the first sign of the development of allergic diseases, including asthma, allergic rhinitis and conjunctivitis [17, 18]. The prevalence of AD ranges from 2.4% to 37% in children and 6.8%–10.6% in adults worldwide [19, 20], and was 12.9% in children aged 1–7 years in China in a previous study [21].

Given the facts that fetuses are more vulnerable to PFASs and PFASs might impair immune function [22], we investigated the effects of prenatal exposure to PFASs on AD in children up to 24 months of age in a prospective cohort study in Shanghai, China.

## Methods

### Study design

A prospective birth cohort study was conducted in two large tertiary-level hospitals in Shanghai from 2012 to 2015. Women were recruited if they had a singleton pregnancy and planned to live in Shanghai for at least 2 years. A written consent was obtained. Trained nurses conducted face-to-face interviews and collected information on age, parental education levels and family income, parental history of atopic diseases (i.e. AD, allergic rhinitis, hives or asthma), alcohol use, smoking status, including secondhand smoke and active smoking during pregnancy. At birth, trained nurses measured newborn’s anthropometric parameters and collected umbilical cord blood. Information was also collected from hospital records with patients’ consent.

A total of 1056 baby-mother pairs were enrolled in the study. Two hundred forty-five children were excluded due to missing data on birthweight (*n* = 3) or loss to follow-up by 24 months old, resulting in 811 (76.8%) for further data analyses.

Cases of AD were defined by the International Study of Asthma and Allergies in Childhood (ISAAC) questionnaire through three questions: “Has your child ever had an itchy rash which was coming and going for at least six months at any time?”, “Has the itchy rash been coming and going over elbows, knees, face, wrists, or generalized (4 or more localizations)?”, and “Has your child ever had atopic dermatitis diagnosed by a doctor?” [23]. Pictures of child AD were used to aid parental reporting. Two dermatologists diagnosed AD independently based on the questionnaires for all the children. Disagreements were resolved by consensus.

### Questionnaire survey

At the 6-month follow-up, an online survey was conducted to collect information on duration of breastfeeding, consumption of fruits, vegetables, egg, wheat, soy bean, and shrimp before 6 months of age, living environment and post-natal environmental smoke exposure. AD related questions from ISAAC were also asked. At 12-month and 24-month follow-up visits, face-to-face interviews were conducted to collect similar information as that in 6-month follow-up visit.

### Sample collection and preparation

Blood samples were collected at delivery by double clamping the umbilical cord at the placental and infant ends. For plasma preparation, cord blood was collected in sterile EDTA tubes and centrifuged (10 min, 2400 g). Plasma was separated and stored at −80 °C until delivery to the research laboratory for PFASs analysis.

### Measurement of PFASs concentration

A total of 10 PFASs were measured, including PFOA, PFOS, perfluorononanoic acid (PFNA), perfluorodecanoic acid (PFDA), perfluoroundecanoic acid (PFUA), PFHxS, perfluorooctane sulfonamide (PFOSA), perfluorododecanoic acid (PFDoA), perfluorobutane sulfonic acid (PFBS) and perfluoroheptanoic acid (PFHpA). The PFASs and internal standard solution (^13^C4-PFOS and ^13^C4-PFOA) were purchased from Wellington Laboratories (Guelph, Ontario, Canada) and Sigma-Aldrich (St. Louis, MO, USA). Purities of all standards were ≥ 95%.

Detailed methods of PFASs measurements have been described in our previous report [24]. Briefly, the frozen samples were firstly thawed at room temperature and then vortex mixed for 15 s to ensure homogeneity. A 100 μL plasma sample contained in a polypropylene centrifuge tube was vortexed with 10 μL of 50 ng/mL internal standard solution for 30s. Then 150 μL of methanol and 150 μL acetonitrile containing 1% formic acid were added to each sample before the second vortex. The mixture was sonicated for 10 min and then centrifuged at 12,000 rpm for 10 min. The supernatant was collected (~ 100 μL) and then filtered through 0.22-μm Nylon syringe filter into a 1.5 mL auto-sampler vial.

The calibration standard solution was prepared in 100 μL of bovine serum and went through sample preparations under the same procedures. The final concentrations for all analytes were 0.5–100 ng/mL containing a fixed amount of internal standard (25 ng/mL) in bovine serum samples. The separation and detection were performed on high performance liquid chromatography with tandem mass spectrometry (HPLC--MS/MS, Agilent 1290–6490, Agilent Technologies Inc., U.S.A). A 2-μL aliquot of the sample extract was injected into a ZORBAX Eclipse Plus C18 column (2.1 × 100 mm, 1.8 μm; Agilent, USA) maintained at 35 °C and equipped with a C18 pre-column (2.1 × 5 mm, 1.8 μm). The flow rate was 0.3 mL/min. Mobile phases A and B were water contained 10 mM ammonium acetate and methanol, respectively. The gradient elution started at 60% methanol and increased to 80% after 5 min, then increased to 90% at 10 min; it was held at 90% for 1 min, and then returned back to 40% methanol at 12 min. The instrument was operated in dynamic multiple reaction monitoring (MRM) with an electrospray ionization (ESI) negative mode.

Recovery rate ranged between 80–110%. Intra- and inter-day coefficient of variations was below 10%. The detection limits for PFASs ranged from 0.009 to 0.12 ng/mL. For concentrations below the detection limits, a value of half the lower limit was assigned. PFOSA and PFHpA were detected in < 30% of the samples, and thus were not used for further analysis. PFOS, PFNA, PFHxS were detected in all samples, and the other 5 PFASs were detectable in > 90% of the samples.

### Statistical analysis

Socio-demographic data and PFASs concentrations in cord blood plasma were compared between children with and without AD, by Wilcoxon Rank Sum test or Cochran-Mantel-Haenszel test, where appropriate. Medians (Q1, Q3) were calculated for PFASs. Because of the skewed distributions, a log transformation was performed for cord blood plasma PFASs concentrations for logistic regression.

The association of log-cord blood plasma PFASs concentrations and AD were analyzed by univariate and multivariate logistic regression models. PFASs concentrations were log transformed in the first model and categorized into quartiles with the lowest quartile as the reference level in the second model. Potential confounders were selected based on a directed acyclic graph, included infant sex, parity (nulliparous and parous), birth weight, gestational age at delivery, mode of delivery, maternal pre-pregnancy BMI, maternal age, maternal education, maternal ethnicity, paternal age, paternal education, parental history of allergic disorders, paternal smoking during pregnancy, family income and breasting during the first 6 months (Additional file 1: Figure S1). As only few women drank (1.98%) and smoked (0.5%), maternal drinking and smoking were not included in the fully adjusted model. All the analyses were performed using the SAS software, version 9.4 (SAS Institute, Inc., Cary, NC, USA).

## Results

A total of 687 children completed a 2-year follow-up visit and had PFASs measurement. During the first 24 months, 173 children (25.2%) developed AD. There were no significant differences between those followed up and the total population with regard to basic characteristics (shown in Additional file 2: Table S1). Table 1 shows that the mean maternal and paternal ages were 29 and 31 years, respectively. The majority of pregnant women had normal weight (18.5 ≤ BMI < 25 kg/m^2^, 70.9%) with median BMI of 21.5 kg/m^2^ before pregnancy**.** Most couples had college education or higher. Only 3 women smoked during pregnancy and 13 consumed alcohol during pregnancy. However, 31.5% of mothers were exposed to paternal smoking during pregnancy. Among the newborns, 357 (52.1%) were boys; 517 (75.4%) were delivered by cesarean section. Mean (± SD) birth weight was 3388 g (± 469) with an average gestational age of 38.8 weeks (± 1.3). The proportion of breastfeeding infants in the AD group was 87.3%, which was slightly higher than that in the non-AD group (78.2%). Univariate analyses show that compared with children without AD, children with AD were more likely to have a father with older age, family history of allergic disorders and without older siblings.

**Table 1:** Characteristics of the study population by childhood atopic dermatitis (AD) status

	All (*N* = 687)	Non-eczema (*N* = 514)	Eczema (*N* = 173)	P^a^
Prevalence of atopic dermatitis	173 (25.2)			
Parental characteristics				
Maternal age (years)	29.3 ± 3.8	29.2 ± 3.9	29.4 ± 3.5	0.82
< 25	48 (7.0)	38 (7.4)	10 (5.8)	
25–30	347 (50.5)	261 (50.8)	86 (49.7)	
30–35	226 (32.9)	165 (32.1)	61 (35.3)	
≥ 35	66 (9.6)	50 (9.7)	16 (9.2)	
Paternal age (years)	31.7 ± 4.5	31.5 ± 4.4	32.1 ± 4.7	0.12
< 25	15 (2.2)	10 (2.0)	5 (2.9)	
25–30	228 (33.2)	173 (33.7)	55 (31.8)	
30–35	294 (42.8)	229 (44.6)	65 (37.6)	
≥ 35	150 (21.8)	102 (19.8)	48 (27.8)	
Maternal pre-pregnancy BMI	21.5 ± 3.6	21.4 ± 3.6	21.8 ± 3.6	0.67
< 18.5	111 (16.2)	87 (16.9)	24 (13.9)	
18.5–25	487 (70.9)	363 (70.6)	124 (71.7)	
25–30	68 (9.9)	50 (9.7)	18 (10.4)	
≥ 30	21 (3.0)	14 (2.7)	7 (4.0)	
Maternal education				
High school or lower	97 (14.1)	78 (15.2)	19 (11.0)	0.38
College	529 (77.0)	390 (75.9)	139 (80.3)	
Postgraduate or higher	61 (8.9)	46 (8.9)	15 (8.7)	
Paternal education				
High school or lower	93 (13.5)	70 (13.6)	23 (13.3)	0.57
College	503 (73.2)	380 (73.9)	123 (71.1)	
Postgraduate or higher	91 (13.3)	64 (12.5)	27 (15.6)	
Family history of allergic disorders				
Yes	143 (20.8)	94 (18.3)	49 (28.3)	0.02
No	533 (77.6)	412 (80.2)	121 (69.9)	
Unknown	11 (1.6)	8 (1.5)	3 (1.7)	
Maternal smoking				0.31
Yes	3 (0.4)	3 (0.6)	0 (0)	
No	679 (99.6)	507 (99.4)	172 (100)	
Parental smoking				
Yes	215 (31.5)	167 (32.7)	48 (28.1)	0.26
No	467 (68.5)	344 (67.3)	123 (71.9)	
Maternal alcohol intake				
Yes	13 (1.9)	12 (2.3)	1 (0.6)	0.15
No	671 (98.1)	501 (97.7)	170 (99.4)	
Newborn characteristics				
Infant sex				
Male	357 (52.1)	260 (50.6)	97 (56.7)	0.16
Female	328 (47.9)	254 (49.4)	74 (43.3)	
Birth weight (g)	3388 ± 469	3377 ± 482	3423 ± 428	0.52
< 2500	18 (2.6)	15 (2.9)	3 (1.7)	
2500–4000	608 (88.5)	451 (87.7)	157 (90.8)	
≥ 4000	61 (8.9)	48 (9.36)	13 (7.5)	
Gestational age (weeks)	38.8 ± 1.3	38.8 ± 1.4	38.8 ± 1.1	0.18
≤ 37	54 (7.9)	45 (8.8)	9 (5.2)	
38	192 (28.0)	139 (27.0)	53 (30.6)	
39	261 (38.0)	187 (36.4)	74 (42.8)	
40	137 (19.9)	110 (21.4)	27 (15.6)	
41	43 (6.3)	33 (6.4)	10 (5.8)	
Mode of delivery				
Caesarean section	517(75.4)	389 (75.7)	128 (74.4)	0.74
Vaginal delivery	169 (24.6)	125 (24.3)	44 (25.6)	
Parity				
Nulliparous	626 (91.2)	462 (89.9)	164 (95.4)	0.03
Parous	60 (8.8)	52 (10.1)	8 (4.6)	
Breastfeeding				
Yes	553 (80.5)	402 (78.2)	151 (87.3)	0.03
No	28 (4.1)	22 (4.3)	6 (3.5)	
Unknown	106 (15.4)	90 (17.5)	16 (9.3)	

Data presented are n (%) or median ± standard deviation

The missing data: maternal smoking (*n* = 5); paternal smoking (*n* = 5); maternal alcohol intake (*n* = 3); infant sex (*n* = 2); mode of delivery (*n* = 1); parity (*n* = 1)

^a^Chi-square test was used to compare the difference of characteristics between Non-AD and AD group

Among the 811 subjects, 124 had insufficient volume of cord blood for laboratory analyses. The median (Q1-Q3) values of PFOA, PFOS, PFNA, PFDA, PFUA, PFDoA, PFHxS and PFBS were 6.98 (4.94–9.55), 2.48 (1.82–3.24), 0.65 (0.50–0.83), 0.36 (0.23–0.54), 0.40 (0.29–0.53), 0.09 (0.07–0.13), 0.16 (0.13–0.20), 0.05 (0.04–0.06) ng/mL, respectively (Table 2). Most PFASs concentrations were slightly but not significantly higher in female than male infants. PFOA concentration in cord blood was higher in children with AD than in children without AD, while no statistically significant difference in other PFASs was found between those with and without AD (Table 3).

**Table 2:** Concentrations (ng/mL) of the most prevalent PFASs in cord plasma samples of this study population

	PFOS	PFOA	PFNA	PFDA	PFUA	PFDoA	PFHxS	PFBS
All (*n* = 687)								
N > LOD (%)	687 (100)	686 (99.9)	687 (100)	681 (99.1)	686 (99.9)	621 (90.4)	687 (100)	668 (97.2)
Range	0.39–65.61	<LOD–29.97	0.18–3.29	<LOD–5.73	<LOD–5.27	<LOD–1.14	0.05–0.85	<LOD–0.46
Mean (SD)	2.93 (3.11)	7.73 (3.98)	0.70 (0.29)	0.45 (0.42)	0.46 (0.34)	0.10 (0.07)	0.18 (0.08)	0.05 (0.03)
Median	2.48	6.98	0.64	0.36	0.4	0.09	0.16	0.05
Female (*n* = 328)								
N > LOD (%)	328 (100)	328 (100)	328 (100)	324 (98.8)	327 (99.7)	291 (88.7)	328 (100)	318 (97.0)
Range	0.39–18.68	0.70–29.97	0.18–3.29	<LOD–2.51	<LOD–2.60	<LOD–0.51	0.07–0.78	<LOD–0.39
Mean (SD)	2.81 (1.86)	7.57 (3.71)	0.71 (0.31)	0.44 (0.32)	0.45 (0.26)	0.11 (0.05)	0.17 (0.08)	0.05 (0.03)
Median	2.47	7	0.66	0.38	0.41	0.1	0.16	0.05
Male (*n* = 357)								
N > LOD (%)	357 (100)	356 (99.7)	357 (100)	355 (99.4)	357 (100)	328 (91.9)	359 (100)	348 (97.5)
Range	0.62–65.61	<LOD–25.99	0.18–2.04	<LOD–5.73	0.05–5.27	<LOD–1.14	0.05–0.85	<LOD–0.46
Mean (SD)	3.04 (3.93)	7.86 (4.22)	0.69 (0.28)	0.45 (0.50)	0.46 (0.40)	0.10 (0.08)	0.18 (0.08)	0.05 (0.03)
Median	2.49	6.89	0.64	0.35	0.39	0.09	0.16	0.05

*LOD* limit of detection, *SD* standard deviation, *CI* confidence intervals

LOD (ng/mL): PFOA (0.09), PFOS (0.09), PFNA (0.02), PFDA (0.02), PFUA (0.02), PFDoA (0.05), PFHxS (0.02), PFBS (0.009)

**Table 3:** Cord blood concentrations of PFASs (ng/mL) by childhood atopic dermatitis (AD) status

PFAS	Non-AD (*N* = 514)	AD (*N* = 173)	P
PFOS	2.46 (1.80–3.17)	2.54 (1.83–3.37)	0.29
PFOA	6.76 (4.84–9.29)	7.17 (5.22–10.19)	0.04
PFNA	0.64 (0.49–0.81)	0.66 (0.54–0.86)	0.09
PFDA	0.36 (0.23–0.52)	0.39 (0.26–0.58)	0.13
PFUA	0.40 (0.29–0.52)	0.41 (0.30–0.57)	0.29
PFDoA	0.09 (0.07–0.13)	0.09 (0.07–0.12)	0.87
PFHxS	0.16 (0.13–0.20)	0.16 (0.14–0.21)	0.16
PFBS	0.05 (0.04–0.06)	0.05 (0.04–0.06)	0.43

Abbreviations: *PFOS* Perfluorooctane sulfonate, *PFNA* Perfluorononanoic acid, *PFDA* Perfluorodecanoic acid, *PFUA* Perfluoroundecanoic acid, *PFHxS* Perfluorohexanesulfonate, *PFOA* Perfluorooctanate, *PFDoDA* Perfluorododecanoic acid, *PFBS* Perfluorobutane sulfonate

Table 4 shows that the highest PFOA quartile was associated with a 1.7-fold increase in AD risk (OR 1.74, 95% CI 1.02–2.95) after adjusting for potential confounders, and the second PFDoA quartile was associated with a 1.8-fold increase in AD risk (OR 1.83, 95% CI 1.09–3.08). The significant modification effect of sex on the associations of interest was found only for the association between PFHxS exposure and AD. Thus, the effects of PFASs on AD were generally similar in male and female children.

**Table 4:** Association between cord blood concentrations of PFASs and risk for childhood AD (*n* = 687)

PFASs	Number	Number of cases	Crude OR (95%CI)	Adjusted OR (95%CI)	p for sex-PFASs interaction
PFOS					
Continuous^a^	687	173	1.17 (0.85–1.62)	1.23 (0.85–1.76)	0.33
Q1 (< 1.81)^b^	171	40	1	1	0.26
Q2 (1.81–2.48)	172	42	1.06 (0.64–1.74)	0.93 (0.56–1.58)	
Q3 (2.48–3.24)	172	41	1.03 (0.62–1.69)	1.00 (0.59–1.70)	
Q4 (≥ 3.24)	172	50	1.34 (0.83–2.18)	1.31 (0.78–2.20)	
p for trend^c^			0.27	0.27	
PFOA					
Continuous^a^	687	173	1.44 (1.02–2.03)	1.35 (0.93–1.97)	0.17
Q1 (< 4.94)^b^	171	34	1	1	0.14
Q2 (4.94–6.98)	172	46	1.47 (0.89–2.44)	1.48 (0.87–2.52)	
Q3 (6.98–9.55)	172	39	1.18 (0.70–1.98)	1.16 (0.67–2.00)	
Q4 (≥ 9.55)	172	54	1.84 (1.12–3.02)	1.74 (1.02–2.95)	
p for trend^c^			0.04	0.1	
PFNA					
Continuous^a^	687	173	1.49 (0.95–2.32)	1.53 (0.94–2.47)	0.46
Q1 (< 0.50)^b^	171	36	1	1	0.33
Q2 (0.50–0.64)	172	44	1.29 (0.78–2.13)	1.15 (0.67–1.97)	
Q3 (0.64–0.83)	172	44	1.29 (0.78–2.13)	1.20 (0.71–2.05)	
Q4 (≥ 0.83)	172	49	1.49 (0.91–2.45)	1.47 (0.87–2.50)	
p for trend^c^			0.13	0.15	
PFDA					
Continuous^a^	687	173	1.18 (0.93–1.50)	1.22 (0.94–1.58)	0.65
Q1 (< 0.23)^b^	171	40	1	1	0.68
Q2 (0.23–0.36)	172	36	0.87 (0.52–1.44)	0.94 (0.55–1.60)	
Q3 (0.36–0.54)	172	45	1.16 (0.71–1.90)	1.15 (0.68–1.95)	
Q4 (≥ 0.54)	172	52	1.42 (0.88–2.30)	1.58 (0.94–2.65)	
p for trend^c^			0.08	0.06	
PFUA					
Continuous^a^	687	173	1.18 (0.86–1.63)	1.24 (0.88–1.75)	0.67
Q1 (< 0.29)^b^	171	40	1	1	0.83
Q2 (0.29–0.40)	172	40	0.99 (0.60–1.64)	1.01 (0.59–1.71)	
Q3 (0.40–0.53)	172	44	1.13 (0.69–1.84)	1.13 (0.67–1.90)	
Q4 (≥ 0.53)	172	49	1.31 (0.80–2.12)	1.36 (0.81–2.28)	
p for trend^c^			0.23	0.22	
PFDoA					
Continuous^a^	687	173	0.96 (0.70–1.30)	1.00 (0.72–1.39)	0.28
Q1 (≤ 0.069)^b^	171	36	1	1	0.76
Q2 (0.069–0.094)	172	54	1.72 (1.05–2.80)	1.83 (1.09–3.08)	
Q3 (0.094–0.125)	172	42	1.21 (0.73–2.01)	1.37 (0.80–2.34)	
Q4 (≥ 0.125)	172	41	1.17 (0.71–1.95)	1.18 (0.68–2.04)	
p for trend^c^			0.93	0.87	
PFHxS					
Continuous^a^	687	173	1.31 (0.80–2.13)	1.08 (0.62–1.85)	0.43
Q1 (≤ 0.13)^b^	171	37	1	1	0.05
Q2 (0.13–0.16)	172	43	1.21 (0.73–1.99)	1.25 (0.74–2.12)	
Q3 (0.16–0.20)	172	46	1.32 (0.81–2.17)	1.15 (0.68–1.94)	
Q4 (≥ 0.20)	172	47	1.36 (0.83–2.23)	1.14 (0.67–1.94)	
p for trend^c^			0.2	0.73	
PFBS					
Continuous^a^	687	173	1.20 (0.87–1.66)	1.22 (0.87–1.72)	0.83
Q1 (≤ 0.037)^b^	170	39	1	1	0.33
Q2 (0.037–0.047)	171	43	1.13 (0.69–1.86)	1.28 (0.75–2.17)	
Q3 (0.047–0.062)	174	46	1.21 (0.74–1.97)	1.18 (0.70–2.00)	
Q4 (≥ 0.062)	172	45	1.19 (0.73–1.95)	1.15 (0.68–1.95)	
p for trend^c^			0.46	0.70	

Models were adjusted for all the confounders followed, maternal age (continuous), maternal pre-pregnancy BMI (continuous), gestational week at delivery (continuous), birth weight (categorical), maternal education (categorical), paternal education (categorical), parity (categorical), mode of delivery (categorical), family history of allergic disorders (categorical), infant sex (categorical), family income (categorical), maternal ethnicity (categorical), paternal smoking (categorical) and breastfeeding (categorical)

^a^Log-transformed PFASs as continuous variables

^b^Reference category

^c^*p*-Values for exposure were modeled according to the median value of each quartile

Table 5 shows that overall, prenatal exposures to PFOA, PFDA, PFDoA and PFHxS were significantly associated with childhood AD in girls during the first 24 months (Table 5). In the first fully adjusted model, a log-unit increase in PFOA was associated with a 2.1-fold increase in AD risk (AOR 2.07, 95% CI 1.13–3.80). The corresponding number for PFNA was 2.22 (1.07–4.58). In the second fully adjusted model, when PFASs were grouped by quartiles, AOR increased with increasing PFOA and PFNA levels. The strongest associations were found in Q4. Specially, the highest quartiles of PFOA, PFNA, PFDA and PFHxS were associated with AD with AOR (95% CI) being 2.52 (1.12–5.68), 2.14 (0.97–4.74), 2.14 (1.00–4.57), and 2.30 (1.03–5.15), respectively. Additionally, the second quartile of perfluorododecanoic acid (PFDoA) was associated with a 3.2-fold increase in AD risk (3.24, 1.44–7.27). Due to small sample size, the associations between AD with prenatal exposure to PFNA were close to statistical significance. No other PFASs were significantly associated with AD in girls. However, no significant associations were found between PFASs and AD in boys (Additional file 2: Table S2).

**Table 5:** Association between cord blood concentrations of PFASs and risk for childhood AD in female children (*n* = 328)

PFASs	Number	Number of cases	Crude OR (95%CI)	Adjusted OR (95%CI)
PFOS				
Continuous^a^	328	74	1.05 (0.64–1.71)	1.10 (0.64–1.87)
Q1 (< 1.80)^b^	82	21	1	1
Q2 (1.80–2.47)	82	16	0.70 (0.34–1.47)	0.73 (0.33–1.61)
Q3 (2.47–3.22)	82	16	0.70 (0.34–1.47)	0.71 (0.32–1.60)
Q4 (≥ 3.22)	82	21	1.00 (0.50–2.02)	1.08 (0.50–2.35)
p for trend^c^			0.85	0.74
PFOA				
Continuous^a^	328	74	2.05 (1.17–3.61)	2.07 (1.13–3.80)
Q1 (< 4.94)^b^	82	13	1	1
Q2 (4.94–7.00)	82	14	1.09 (0.48–2.50)	1.23 (0.52–2.93)
Q3 (7.00–9.42)	82	20	1.71 (0.79–3.73)	1.81 (0.79–4.14)
Q4 (≥ 9.42)	82	27	2.61 (1.23–5.52)	2.52 (1.12–5.68)
p for trend^c^			0.004	0.01
PFNA				
Continuous^a^	328	74	1.92 (0.99–3.74)	2.22 (1.07–4.58)
Q1 (< 0.50)^b^	82	15	1	1
Q2 (0.50–0.66)	82	15	1.00 (0.45–2.21)	1.10 (0.46–2.62)
Q3 (0.66–0.85)	82	19	1.35 (0.63–2.88)	1.30 (0.57–2.97)
Q4 (≥ 0.85)	82	25	1.96 (0.94–4.07)	2.14 (0.97–4.74)
p for trend^c^			0.04	0.05
PFDA				
Continuous^a^	328	74	1.14 (0.80–1.63)	1.25 (0.86–1.84)
Q1 (< 0.25)^b^	82	19	1	1
Q2 (0.25–0.38)	82	17	0.87 (0.41–1.82)	1.01 (0.46–2.23)
Q3 (0.38–0.53)	82	12	0.57 (0.26–1.26)	0.54 (0.22–1.30)
Q4 (≥ 0.53)	82	26	1.54 (0.77–3.08)	2.14 (1.00–4.57)
p for trend^c^			0.35	0.13
PFUA				
Continuous^a^	328	74	1.15 (0.71–1.86)	1.24 (0.74–2.10)
Q1 (< 0.29)^b^	82	17	1	1
Q2 (0.29–0.41)	82	17	1.00 (0.47–2.13)	1.11 (0.49–2.50)
Q3 (0.41–0.54)	82	19	1.15 (0.55–2.42)	1.20 (0.55–2.64)
Q4 (≥ 0.54)	82	21	1.32 (0.64–2.73)	1.55 (0.70–3.43)
p for trend^c^			0.41	0.28
PFDoA				
Continuous^a^	328	74	0.88 (0.57–1.36)	0.91 (0.57–1.46)
Q1 (≤ 0.069)^b^	82	13	1	1
Q2 (0.069–0.096)	82	27	2.61 (1.23–5.52)	3.24 (1.44–7.27)
Q3 (0.096–0.128)	82	17	1.39 (0.63–3.08)	1.44 (0.62–3.36)
Q4 (≥ 0.128)	82	17	1.39 (0.63–3.08)	1.63 (0.68–3.92)
p for trend^*c*^			0.91	0.74
PFHxS				
Continuous^a^	328	74	1.59 (0.77–3.31)	1.43 (0.64–3.15)
Q1 (≤ 0.13)^b^	82	14	1	1
Q2 (0.13–0.16)	82	16	1.18 (0.53–2.60)	1.43 (0.62–3.30)
Q3 (0.16–0.20)	82	18	1.37 (0.63–2.97)	1.29 (0.55–2.99)
Q4 (≥ 0.20)	82	26	2.26 (1.08–4.73)	2.30 (1.03–5.15)
p for trend^c^			0.03	0.06
PFBS				
Continuous^a^	328	74	1.22 (0.75–1.97)	1.23 (0.74–2.04)
Q1 (≤ 0.037)^b^	82	14	1	1
Q2 (0.037–0.047)	81	18	1.39 (0.64–3.02)	1.65 (0.70–3.84)
Q3 (0.047–0.061)	83	23	1.86 (0.88–3.94)	1.83 (0.81–4.17)
Q4 (≥ 0.061)	82	19	1.46 (0.68–3.17)	1.50 (0.65–3.48)
p for trend^c^			0.25	0.35

Models were adjusted for all the confounders followed, maternal age (continuous), maternal pre-pregnancy BMI (continuous), gestational week at delivery (continuous), birth weight (categorical), maternal education (categorical), paternal education (categorical), parity (categorical), mode of delivery (categorical), family history of allergic disorders (categorical), family income (categorical), maternal ethnicity (categorical), paternal smoking (categorical) and breastfeeding (categorical)

^a^Log-transformed PFASs as continuous variables

^b^Reference category

^c^p-Values for exposure were modeled according to the median value of each quartile

## Discussion

Our prospective cohort study suggests that prenatal PFASs exposures may increase the risk of childhood AD. In particular, we found that PFOA, PFDA, PFDoA and PFHxS in cord blood increased the risk of childhood AD in girls only. In addition, the associations between AD with prenatal exposure to PFNA were close to statistical significance.

Five studies have reported the association between prenatal exposure and childhood AD, but the results were inconsistent. In a cohort study conducted in Taiwan, the highest quartile of PFOS appeared to be positively associated with AD with an OR of 2.79 (95% CI: 1.06, 7.38) [25]. However, after adjusted by potential confounders, the association was no longer statistically significant. Another two birth cohort studies from Norway and Japan found no significant associations between PFOA, PFOS, PFNA and PFHxS and AD or eczema in early childhood [26, 27]. Likewise, a birth cohort study in 1024 infants conducted in Greenland and Ukraine, reported that principal component (PC) score, dominated by PFOA, was not associated with eczema [28]. In contrast, a birth cohort study in Japan involving 2063 subjects found that prenatal exposure to perfluorotridecanoic acid (PFTrDA) was associated with a decreased risk of developing eczema in female infants at 24 months (OR = 0.39; 95% CI: 0.23, 0.64) [29]. After following for another 2 years, PFOS levels in the highest quartile were associated with an increased risk of total infectious diseases in all children (OR = 1.61; 95% CI: 1.18, 2.21), while the highest quartile of PFHxS was associated with a higher risk of total infectious diseases only among girls (OR = 1.55, 95% CI: 0.976, 2.45) [30]. These studies provided evidence for the potential long-lasting effect of PFASs on children’s immune system.

Several studies also reported associations between PFASs and other allergic disorders in children and adults. A US national study found that serum PFOA was positively associated with asthma (OR = 1.18, 95% CI: 1.01, 1.39) in adolescents aged 12–19 years, while PFOS was inversely associated with both asthma and wheezing (OR = 0.88; 95% CI: 0.74, 1.04, and OR = 0.83; 95% CI: 0.67, 1.02, respectively) [15]. In addition, a case-control study with 231 asthmatic and 225 non-asthmatic children aged 10–15 years in Taiwan found that PFOA and PFOS were associated with asthma in a dose-response pattern [14].

The mechanisms of prenatal PFASs exposure on the development of AD have not been fully understood. Animal and human studies suggest that PFOA and PFOS may increase IgE levels in blood. For example, PFASs concentrations in cord blood were positively associated with cord blood IgE levels in Taiwan infants (per ln-unit: β = 0.134 KU/l, *p* = 0.047 for PFOA; β = 0.161 KU/l, *p* = 0.017 for PFOS) [25]. Furthermore, both animal and human studies suggested that PFOS may increase Th2 cytokines secretion (IL-4 and IL-10) and decrease Th1 cytokines (IL-2 and IFN-γ) at doses comparable to those in humans, shifting immune response toward Th2 state [31, 32]. A previous study showed that PFOA, PFOS and PFDA suppressed LPS-induced TNF-α production; PFOS and PFDA inhibited PHA-induced IL-10 release in human peripheral blood leukocytes (hPBL) and human promyelocytic cell line THP-1 [33].

Oxidative stress might be another potential mechanism of the immunotoxic effects of PFASs. Wielsøe et al. exposed human hepatoma cell line (HepG2) with PFHxS, PFOA, PFOS and PFNA for 24 h and found a dose-dependent increase in DNA damage and increased ROS generation [34]. PFNA was also found to have immunomodulatory effects on leukocyte populations and immune function, including decreased spleen size and a decreased ratio of CD4+/CD8+ double-positive population in thymus of mice [35].

In addition, peroxisome proliferator activated receptors (PPARs) signaling pathway might also play a role in the effects of PFASs on childhood AD. Previous study showed that PFOA and PFOS significantly increased PPAR-α and PPAR-γ in human cells [36], which, in turn, could shift cytokine secretion by inhibiting IFN-γ and promoting IL-4 secretion in human T-cell lines [37, 38]. The changes of these cytokines resulted from PFASs exposure might impair the immune system and immune responses, especially in young children when the immune system is still developing.

Nonetheless, a prospective cohort study in Japan found an inverse association between maternal PFOS concentration and cord blood IgE levels among female infants (β = −3.08, 95%CI: -5.43, −0.73) [26]. Thus, the evidence is far from consistent, and our understanding of the underlying biological mechanisms is still incomplete.

A birth cohort study conducted in Japan between 2002 and 2005, showed that cortisol and cortisone concentrations were significantly lower in cord blood of infants with prenatal PFOS exposure in Q4 compared with those in Q1 (β = −23.98 ng/mL, 95% CI: -47.12, −11.99 for cortisol; β = −63.21 ng/mL, 95% CI: -132.56, −26.72 for cortisone). Meanwhile, the highest quartile of prenatal PFOS exposure was positively associated with a significant higher DHEA level (β = 1.33 ng/mL, 95% CI: 0.17, 1.82), whereas PFOA showed a negative association with DHEA levels (β = −1.23 ng/mL, 95% CI: -1.72, −0.25) [39]. In addition, animal and human data suggested that perinatal exposure to glucocorticoid may program the fetal hypothalamic-pituitary-adrenal (HPA) axis affecting its development, resulting in long-lasting effect in HPA axis function [40, 41]. These findings revealed that prenatal exposure may have an effect on the balance of steroid hormones, which was one of the potential mechanisms of the immunotoxical effects of PFASs [42].

Our study discovered that the association between prenatal exposure to PFASs and childhood AD existed only in girls, but the biologic mechanism is unclear. A previous study showed that exposure to higher levels of PFASs resulted in lower testosterone and higher estradiol levels, and more significant associations of PFASs with reproductive hormones were found in male adolescents than in females [43]. This effect was supported by an experimental study conducted in mice [44]. A case-control study reported that PFASs were positively associated with estradiol levels and negatively associated with testosterone levels among asthmatics [45]. Furthermore, the interactions between estradiol and PFASs were significant for PFOS (*p* = 0.026) and PFNA (*p* = 0.043) among girls, suggesting that reproductive hormones might amplify the association between PFASs and asthma.

The PFOA level in our study was higher than that reported in other countries and was the most abundant among all the PFAS members [24]. The higher PFAS levels in Shanghai may be due to higher levels of pollutants in Yangtze river, probably because there are many fluoropolymer factories in the Yangtze Delta. For example, PFAS levels in tap water in Shanghai was higher than that in 19 cities in China, Japan, India, the USA, and Canada between 2006 and 2008 [46].

Our study also showed a very high cesarean section rate. This is consistent with the situation in China where cesarean delivery on maternal request accounts for nearly 50% of all cesarean sections [47]. The effect of cesarean section on AD has not been well studied and the results were inconclusive. The effect of cesarean section on AD during the first 3 years of life was not significant (OR 1.35; 95% CI 0.74–2.47) in a birth cohort study [48]. The OR of having AD in adolescents aged from 12 to 18 years born by caesarean section compared with vaginal delivery was 1.50 (95% CI 1.01–2.22) in a cross-sectional study [49]. However, our study did not show any association (OR 0.94, 95% CI 0.63–1.39).

The prospective cohort study design, the AD diagnosis confirmed by two dermatologists, and the reliable laboratory assessments were strengths of our study. We also adjusted for a number of potential confounders in the multivariable models. On the other hand, it is worth noting that 23.2% of children were excluded from analyses due to missing data or loss to follow-up, resulting in reduced statistical power for sex-stratified analyses. However, there were no significant differences in basic characteristics between those remained in analyses and those excluded. In addition, our study is still not large enough to examine some PFASs with a wider variation of exposure and AD. Finally, unmeasured confounding is possible and residual confounding could not be ruled out.

## Conclusion

Prenatal exposure to PFOA, PFDA, PFDoA and PFHxS was positively associated with childhood AD in girls during the first 24 months of life. In addition, the associations between AD with prenatal exposure to PFNA were close to statistical significance. Future studies are warranted to investigate the underlying mechanisms of the effects of PFASs and allergic diseases in early childhood.

## Additional files

Additional file 1: Figure S1. Direct Acyclic Graph (DAG) of the association between prenatal PFASs exposure and childhood AD. (DOCX 305 kb)

Additional file 2: Table S1. Characteristics of the study population between the current study and total population. **Table S2.** Association between cord blood concentrations of PFASs and risk for childhood AD in male children (*n* = 359). (DOCX 23 kb)

## Abbreviations

AD: Atopic dermatitis
CI: Confidence interval
ESI: Electrospray ionization
HPLC: High performance liquid chromatography
ISAAC: International Study of Asthma and Allergies in Childhood
MRM: Multiple reaction monitoring
OR: Odds ratio
PFASs: Perfluoroalkyl and polyfluoroalkyl substances
PFBS: Perfluorobutane sulfonic acid
PFDA: Perfluorodecanoic acid
PFDoA: Perfluorododecanoic acid
PFHpA: Perfluoroheptanoic acid
PFHxS: Perfluorohexane sulfonic acid
PFNA: Perfluorononanoic acid
PFOA: Perfluorooctanoic acid
PFOS: Perfluorooctane sulfonate
PFOSA: Perfluorooctane sulfonamide
PFUA: Perfluoroundecanoic acid
