# Supplementary material for: Prenatal exposure to perfluoroalkyl and polyfluoroalkyl substances and childhood atopic dermatitis: a prospective birth cohort study
Source: Environ Health. 2018 Jan 17;17:8. doi: 10.1186/s12940-018-0352-7 (PMC5773146; doi:10.1186/s12940-018-0352-7)
Supplement: Supplementary file 1 — Direct Acyclic Graph (DAG) of the association between prenatal PFASs exposure and childhood AD. (DOCX 305 kb) [file 12940_2018_352_MOESM1_ESM.docx]

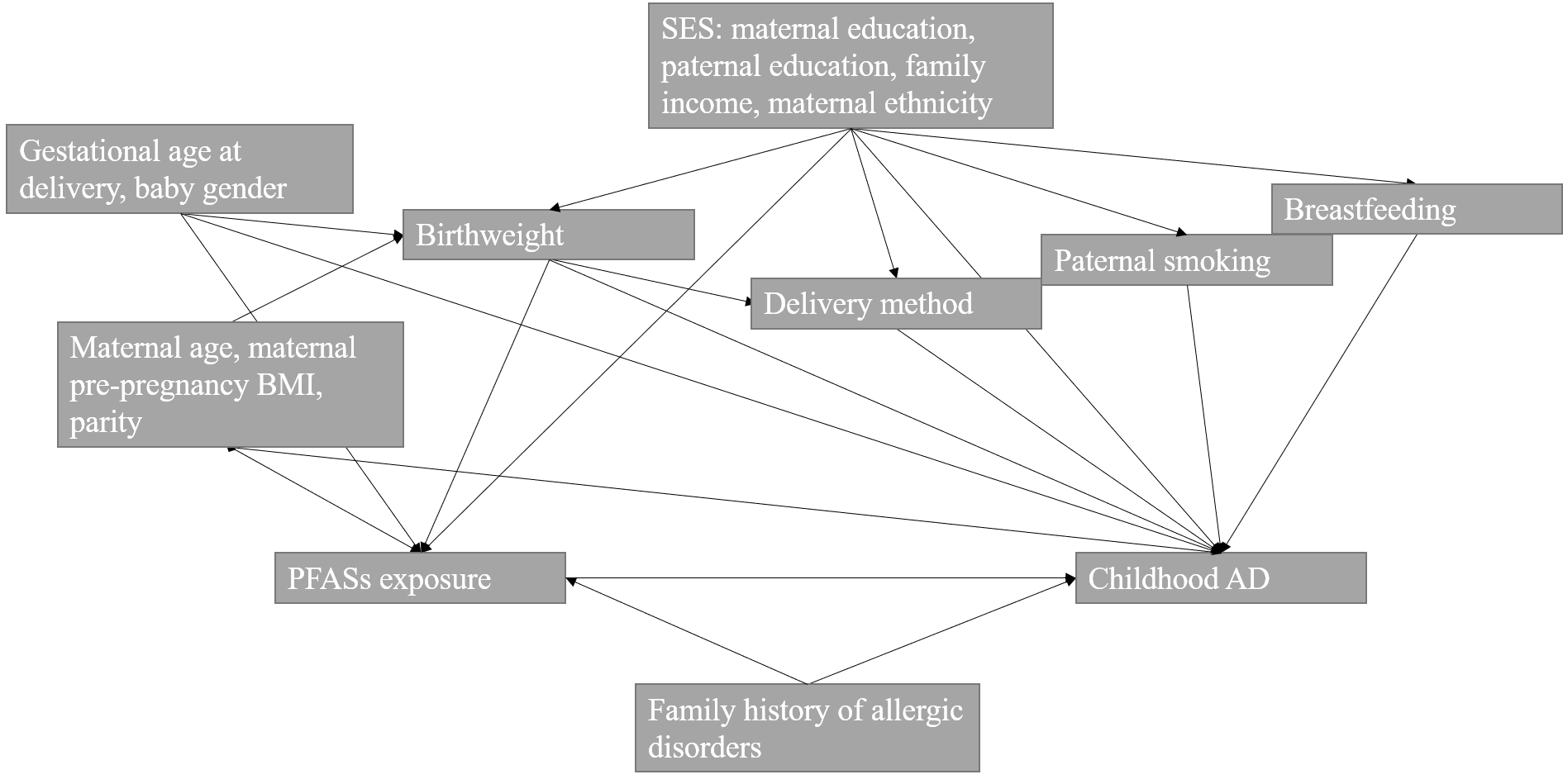


**Figure S1. Direct Acyclic Graph (DAG) of the association between prenatal PFASs exposure and childhood AD**
