# Supplementary material for: Prenatal exposure to perfluoroalkyl and polyfluoroalkyl substances and childhood atopic dermatitis: a prospective birth cohort study
Source: Environ Health. 2018 Jan 17;17:8. doi: 10.1186/s12940-018-0352-7 (PMC5773146; doi:10.1186/s12940-018-0352-7)
Supplement: Supplementary file 2 — Characteristics of the study population between the current study and total population. Table S2. Association between cord blood concentrations of PFASs and risk for childhood AD in male children (n = 359). (DOCX 23 kb) [file 12940_2018_352_MOESM2_ESM.docx]

Table S1. Characteristics of the study population by childhood atopic dermatitis (AD) status during the first 2 years

|  | Current study sample (n=687) | Larger birth cohort (n=1053) | p^#^ |
| --- | --- | --- | --- |
| **Parental characteristics** |  |  |  |
| **Maternal age (years)** | 29.3 ±3.8 | 29.2 ±3.6 | 0.66 |
| <25 | 48 (7.0) | 70 (6.6) |  |
| 25-30 | 347 (50.5) | 539 (51.2) |  |
| 30-35 | 226 (32.9) | 360 (34.2) |  |
| ≥35 | 66 (9.6) | 84 (8.0) |  |
| **Paternal age (years)** | 31.7 ±4.5 | 31.7 ±4.6 | 0.56 |
| <25 | 15 (2.2) | 34 (3.2) |  |
| 25-30 | 228 (33.2) | 333 (31.6) |  |
| 30-35 | 294 (42.8) | 461 (43.8) |  |
| ≥35 | 150 (21.8) | 225 (21.4) |  |
| **Maternal pre-pregnancy BMI** | 21.5 ±3.6 | 21.4 ±3.5 | 0.94 |
| <18.5 | 111 (16.2) | 166 (15.8) |  |
| 18.5-25 | 487 (70.9) | 760 (72.2) |  |
| 25-30 | 68 (9.9) | 98 (9.3) |  |
| ≥30 | 21 (3.0) | 29 (2.7) |  |
| **Maternal education** |  |  | 0.99 |
| High school or lower | 97 (14.1) | 147 (14.0) |  |
| College | 529 (77.0) | 813 (77.3) |  |
| Postgraduate or higher | 61 (8.9) | 92 (8.7) |  |
| **Paternal education** |  |  | 0.69 |
| High school or lower | 93 (13.5) | 142 (13.5) |  |
| College | 503 (73.2) | 786 (74.6) |  |
| Postgraduate or higher | 91 (13.3) | 125 (11.9) |  |
| **Family history of allergic disorders** | | | 0.62 |
| Yes | 143 (20.8) | 193 (18.3) |  |
| No | 533 (77.6) | 846 (80.4) |  |
| Unknown | 11 (1.6) | 14 (1.3) |  |
| **Maternal smoking** |  |  | 0.41 |
| Yes | 3 (0.4) | 3 (0.3) |  |
| No | 679 (99.6) | 1044 (99.6) |  |
| **Parental smoking** |  |  | 0.27 |
| Yes | 215 (31.5) | 335 (32.1) |  |
| No | 467 (68.5) | 708 (67.7) |  |
| **Maternal alcohol intake** | 0 (0) | 2 (0.2) |  |
| Yes |  |  | 0.41 |
| No | 13 (1.9) | 18 (1.7) |  |
| **Newborn characteristics** | 671 (98.1) | 1030 (98.2) |  |
| **Infant sex** |  |  |  |
| Male |  |  | 0.79 |
| Female | 357 (52.1) | 540 (51.5) |  |
| **Birth weight (g)** | 328 ±47.9 | 509 ±48.5 |  |
| <2500 | 3388 (469) | 3400 (460) | 0.99 |
| 2500-4000 | 18 (2.6) | 28 (2.7) |  |
| ≥4000 | 608 (88.5) | 930 (88.3) |  |
| **Gestational age (weeks)** | 61 ±8.9 | 95 ±9.0 |  |
| ≤37 | 38.8 (1.3) | 38.8 (1.3) | 0.78 |
| 38 | 54 (7.9) | 100 (9.5) |  |
| 39 | 192 (28.0) | 303 (28.8) |  |
| 40 | 261 (38.0) | 386 (36.6) |  |
| 41 | 137 (19.9) | 200 (19.0) |  |
| **Mode of delivery** | 43 (6.3) | 64 (6.1) |  |
| Caesarean section |  |  | 0.6 |
| Vaginal delivery | 517(75.4) | 803 (76.5) |  |
| **Parity** | 169 (24.6) | 247 (23.5) |  |
| Nulliparous |  |  | 0.24 |
| Parous | 626 (91.2) | 940 (89.5) |  |

Data presented are n (%) or median ±standard deviation.

^#^ Chi-square test was used to compare the difference of characteristics between Non-AD and AD group.

The missing data: maternal smoking (n =5); paternal smoking (n =8); maternal alcohol intake (n =4); infant sex (n =4); mode of delivery (n =3); parity (n =3).

**Table S2**. Association between cord blood concentrations of PFASs and risk for childhood AD in female children (n=328)

| PFASs | N | Number of cases | Crude OR (95%CI) | Adjusted OR (95%CI) |
| --- | --- | --- | --- | --- |
| **PFOS** |  |  |  |  |
| Continuous ***^a^*** | 359 | 99 | 1.29 (0.84-2.00) | 1.42 (0.84-2.42) |
| Q1 (<1.80) ***^b^*** | 89 | 19 | 1 | 1 |
| Q2 (1.80-2.47) | 91 | 27 | 1.55 (0.79-3.06) | 1.34 (0.64-2.80) |
| Q3 (2.47-3.22) | 89 | 24 | 1.36 (0.68-2.71) | 1.30 (0.61-2.75) |
| Q4 (≥3.22) | 90 | 29 | 1.75 (0.89-3.43) | 1.65 (0.79-3.41) |
| p for trend ***^c^*** |  |  | 0.16 | 0.2 |
| **PFOA** |  |  |  |  |
| Continuous ***^a^*** | 359 | 99 | 1.12 (0.73-1.70) | 0.98 (0.58-1.64) |
| Q1 (<4.94) ***^b^*** | 89 | 21 | 1 | 1 |
| Q2 (4.94-7.00) | 89 | 30 | 1.65 (0.85-3.18) | 1.57 (0.76-3.23) |
| Q3 (7.00-9.42) | 90 | 20 | 0.93 (0.46-1.86) | 0.81 (0.37-1.78) |
| Q4 (≥9.42) | 91 | 28 | 1.44 (0.74-2.79) | 1.34 (0.64-2.82) |
| p for trend ***^c^*** |  |  | 0.63 | 0.83 |
| **PFNA** |  |  |  |  |
| Continuous ***^a^*** | 359 | 99 | 1.17 (0.64-2.14) | 1.17 (0.59-2.30) |
| Q1 (<0.50) ***^b^*** | 89 | 22 | 1 | 1 |
| Q2 (0.50-0.66) | 90 | 27 | 1.31 (0.68-2.53) | 1.13 (0.54-2.35) |
| Q3 (0.66-0.85) | 89 | 25 | 1.19 (0.61-2.32) | 1.16 (0.57-2.38) |
| Q4 (≥0.85) | 91 | 25 | 1.15 (0.59-2.25) | 1.10 (0.53-2.29) |
| p for trend ***^c^*** |  |  | 0.77 | 0.72 |
| **PFDA** |  |  |  |  |
| Continuous ***^a^*** | 359 | 99 | 1.19 (0.86-1.65) | 1.23 (0.84-1.79) |
| Q1 (<0.25) ***^b^*** | 89 | 21 | 1 | 1 |
| Q2 (0.25-0.38) | 89 | 20 | 0.94 (0.47-1.89) | 1.17 (0.54-2.48) |
| Q3 (0.38-0.53) | 90 | 31 | 1.70 (0.88-3.27) | 1.56 (0.76-3.22) |
| Q4 (≥0.53) | 91 | 27 | 1.37 (0.70-2.66) | 1.58 (0.76-3.29) |
| p for trend ***^c^*** |  |  | 0.15 | 0.14 |
| **PFUA** |  |  |  |  |
| Continuous ***^a^*** | 359 | 99 | 1.18 (0.77-1.82) | 1.29 (0.78-2.13) |
| Q1 (<0.29) ***^b^*** | 89 | 23 | 1 | 1 |
| Q2 (0.29-0.41) | 89 | 23 | 1.00 (0.51-1.96) | 1.07 (0.51-2.25) |
| Q3 (0.41-0.54) | 90 | 25 | 1.10 (0.57-2.14) | 1.01 (0.48-2.10) |
| Q4 (≥0.54) | 91 | 28 | 1.28 (0.67-2.45) | 1.37 (0.67-2.82) |
| p for trend ***^c^*** |  |  | 0.43 | 0.45 |
| **PFDoA** |  |  |  |  |
| Continuous ***^a^*** | 359 | 99 | 1.03 (0.67-1.59) | 1.17 (0.72-1.91) |
| Q1 (≤0.069) ***^b^*** | 89 | 23 | 1 | 1 |
| Q2 (0.069-0.096) | 89 | 28 | 1.32 (0.69-2.53) | 1.25 (0.60-2.63) |
| Q3 (0.096-0.128) | 90 | 25 | 1.10 (0.57-2.14) | 1.35 (0.65-2.81) |
| Q4 (≥0.128) | 91 | 23 | 0.97 (0.50-1.90) | 1.00 (0.48-2.07) |
| p for trend ***^c^*** |  |  | 0.79 | 0.88 |
| **PFHxS** |  |  |  |  |
| Continuous ***^a^*** | 359 | 99 | 1.10 (0.57-2.13) | 0.77 (0.35-1.67) |
| Q1 (≤0.13) ***^b^*** | 88 | 23 | 1 | 1 |
| Q2 (0.13-0.16) | 91 | 27 | 1.19 (0.62-2.29) | 1.00 (0.48-2.06) |
| Q3 (0.16-0.20) | 90 | 29 | 1.34 (0.70-2.57) | 1.15 (0.56-2.36) |
| Q4 (≥0.20) | 90 | 20 | 0.81 (0.41-1.61) | 0.55 (0.26-1.20) |
| p for trend ***^c^*** |  |  | 0.66 | 0.21 |
| **PFBS** |  |  |  |  |
| Continuous ***^a^*** | 359 | 99 | 1.14 (0.73-1.78) | 1.21 (0.75-1.94) |
| Q1 (≤0.037) ***^b^*** | 88 | 25 | 1 | 1 |
| Q2 (0.037-0.047) | 88 | 23 | 0.89 (0.46-1.73) | 1.13 (0.54-2.36) |
| Q3 (0.047-0.061) | 92 | 24 | 0.89 (0.46-1.72) | 0.89 (0.44-1.84) |
| Q4 (≥0.061) | 91 | 27 | 1.06 (0.56-2.03) | 1.02 (0.50-2.09) |
| p for trend ***^c^*** |  |  | 0.86 | 0.87 |

Models were adjusted for all the confounders followed, maternal age (continuous), maternal pre-pregnancy BMI (continuous), gestational week at delivery (continuous), birth weight (categorical), maternal education (categorical), paternal education (categorical), parity (categorical), mode of delivery (categorical), family history of allergic disorders (categorical), family income (categorical), maternal ethnicity (categorical), paternal smoking (categorical) and breastfeeding (categorical).

***^a^*** Log-transformed PFASs as continuous variables.

***^b^*** Reference category.

***^c^*** p-Values for exposure were modeled according to the median value of each quartile.
